# The psychological impact of diagnosis of sinonasal, nasopharyngeal, and laryngeal cancers: a systematic review

**DOI:** 10.3389/fpsyg.2024.1355186

**Published:** 2024-07-15

**Authors:** Michela Bonafede, Angelo d'Errico, Sabrina Rugarli, Carolina Mensi, Lucia Miligi, Roberto Calisti, Rosa della Gatta, Sara Piro, Paola Melis, Donatella Vasselli, Alessandra Binazzi

**Affiliations:** ^1^Occupational and Environmental Medicine, Epidemiology and Hygiene Department, Italian Workers' Compensation Authority (INAIL), Roma, Italy; ^2^Epidemiology, Local Health Unit ASL TO3, Piedmont Region, Grugliasco, Italy; ^3^Occupational Health Unit, Fondazione IRCCS Ca' Granda Ospedale Maggiore Policlinico, Milano, Italy; ^4^Sinonasal Cancer Registry of Tuscany, Occupational and Environmental Epidemiology Unit, Institute for Cancer Research, Prevention and Clinical Network (ISPRO), Firenze, Italy; ^5^Sinonasal Cancer Registry of Marche, Department of Prevention, Unit of Workplace Prevention and Safety and of Occupational Epidemiology (SPreSAL Epi Occ), Macerata Health Authority, Civitanova Marche, Italy

**Keywords:** head and neck cancer, occupational cancer, psychological distress, anxiety, depression, quality of life

## Abstract

**Objective:**

High risk of degraded quality of life and psychological distress is observed in patients diagnosed with sinonasal, nasopharyngeal, and laryngeal cancers, which could be caused by exposure to carcinogens in workplaces. This review aimed to investigate the psychological impact of diagnosis associated with the possible occupational etiology of such neoplasms and to explore the instruments that evaluate the quality of life (QoL), anxiety, and depression in these patients.

**Methods:**

Studies were considered for the review only if they described aspects of the psychological impact of the diagnosis of sinonasal, nasopharyngeal, and laryngeal cancers and reported results distinguished by the tumor site. The psychological impact was assessed in terms of health-related QoL, anxiety, and depression using reliable psychometric questionnaires administered at the time of diagnosis and 1 year later.

**Results:**

In more than 5,900 records identified, 442 studies fulfilled the eligibility criteria and 436 were excluded after full-text screening, resulting in six studies to be finally included in the review. The EORTC Core Quality of Life questionnaire (EORTC QLQ-C30), EORTC QLQ-H&N35, and Functional Assessment of Cancer Therapy (FACT) were used to evaluate the QoL, whereas the Hospital Anxiety and Depression Scale (HADS) and Center for Epidemiologic Studies Depression Scale (CES-D) questionnaires were employed to assess anxiety and depression. QoL scores were similar to those of the general population at the time of diagnosis and remained unchanged or slightly improved at 1 year since diagnosis. In contrast, a higher prevalence of anxiety and depression was observed compared with the general population, although the results were inconsistent across the very few studies identified. No study investigating the association between the potential occupational etiology and QoL or distress was found.

**Conclusion:**

Exploring the existing scientific literature on emotional distress in sinonasal, nasopharyngeal, and laryngeal cancer patients was prompted by concerns over the disfiguring nature of treatment and the additional emotional burden resulting from their occupational etiology. Unfortunately, neither a crucial element nor other risk factors (lifestyle, smoking, drinking, etc.) were examined in any study. Patients' employment history should be considered in order to assess the possible impact of believing they are affected by an occupational exposure disease in the evaluation of their psychological distress. This history would support evidence-based recommendations about dedicated psychological interventions.

## 1 Introduction

Head and neck cancers are the seventh most common cancer globally, accounting for more than 660,000 new cases and 325,000 deaths annually (Sung et al., 2021). Several risk factors are associated with head and neck cancers, including tobacco smoking, alcohol consumption, human papillomavirus (type 16), and Epstein–Barr virus infection [International Agency for Research on Cancer (IARC), 2007; Warnakulasuriya, 2009], along with a broad spectrum of occupational exposures (Paget-Bailly et al., 2012a,b).

These cancers pose a significant clinical and social challenge due to their potential to compromise the delicate functions of crucial organs, with a marked impact on every aspect of a patient's quality of life (QoL). Head and neck cancers (HNCs) are particularly devastating due to the localization and visibility (Fingeret, 2010; Kangas et al., 2013; Fingeret et al., 2014). Patients not only suffer from a potentially lethal disease but also must cope with inevitable and sometimes drastic changes in their appearance, as well as the impairment of certain important and basic abilities. HNC patients may risk enduring permanent or temporary physical disfigurements and total or partial loss of some basic functions such as swallowing, chewing, breathing, and communicating, forcing them to adapt laboriously to a radically transformed internal and external reality. Consequently, HNC patients may experience psychological symptoms such as uncertainty, anxiety, depression, suicidal thoughts, feelings of worthlessness, irritability, fear of recurrence, and feelings of inferiority (Pruyn et al., 1986; Lang et al., 2013; Jimenez-Labaig et al., 2024).

These psychological effects can also impact patients' ability to work, particularly when the emotional consequences of a diagnosis emanate from occupational exposure. This situation is commonly faced by patients with certain types of head and neck cancers, such as sinonasal cancer (SNC), nasopharyngeal carcinoma (NPC), and larynx cancer.

According to the International Agency for Research on Cancer (IARC), there is sufficient evidence that SNCs are causally related to exposure to wood dust, leather dust, and nickel compounds as well as to working in the isopropyl alcohol manufacturing industry, which involves the use of strong acids. However, the evidence indicating a causal association for exposure to hexavalent chromium compounds, formaldehyde, and working in the carpentry, joinery, and textile manufacturing industry is limited [International Agency for Research on Cancer (IARC), 2023].

Occupational exposures to formaldehyde and wood dust have been classified by the IARC as potent carcinogenic agents with sufficient evidence of also causing NPC in humans [International Agency for Research on Cancer (IARC), 2012]. The IARC classified the following occupational agents as carcinogens for laryngeal cancer: asbestos (all forms), strong inorganic acid mists (with sufficient evidence of carcinogenicity), sulfur mustard and occupational exposure to hard bitumens and their emissions during mastic asphalt work and roofing, and working in the rubber manufacturing industry (with limited evidence) (Cogliano et al., 2011).

The role of occupational exposure as a determinant of the psychological impact of diagnosis of SNC, NPC, and laryngeal cancer in patients has been largely disregarded. Asbestos exposure primarily causes mesothelioma, making it a significant occupational hazard. Following mesothelioma, SNC is considered the second major disease caused by occupational exposure to asbestos, characterized by a high work-related attributable fraction (estimated to be in the range of 20%−46%) (Rushton et al., 2012; Slack et al., 2012). In Italy, SNC is monitored through a surveillance system that utilizes data regarding clinical and occupational exposure histories. These data are collected using standardized questionnaires, which is then recorded in the national registry (“Registro Nazionale Tumori Naso-Sinusali: ReNaTuNS”). A first report showed that 63% of SNCs were likely attributable to exposure to occupational hazards (Binazzi et al., 2018).

The network of ReNaTuNS interviewers has found that many contacted patients often refuse to be interviewed due to their disfigured faces or difficulties in speech. Therefore, a decision was made to explore the existing scientific literature on emotional distress in these patients. The research was extended to also include NPC and larynx cancer, which are also frequently caused by exposure to occupational agents, because a former experience with mesothelioma cancer patients had suggested an additional emotional burden due to their occupational etiology (Bonafede et al., 2020). Indeed, interviews of patients affected by mesothelioma have revealed that they face substantial physical and psychological difficulties resulting from the diagnosis of mesothelioma, which is recognized as a traumatic experience associated with depression and despair (Bonafede et al., 2020).

In this context, the purpose of the present study was to conduct a literature review to examine the emotional impact of receiving a diagnosis of SNC, NPC, and laryngeal cancer. Specifically, it sought to assess whether the impact differs according to their occupational etiology and to evaluate the instruments utilized for assessing the impact on the quality of life and psychological distress, with the goal of indentifying a suitable instrument to be employed in interviews with SNC patients contacted by the ReNaTuNS network interviewers.

## 2 Methods

We applied the Navigation Guide methodology for conducting systematic reviews in environmental and occupational health as our guiding methodological framework, wherever feasible. This methodology incorporates established systematic review methods from clinical medicine, such as the standard Cochrane methods for systematic reviews of interventions, to the field of environmental and occupational health to ensure systematic and rigorous synthesis of evidence on environmental and occupational risk factors, which reduces bias and maximizes transparency (Woodruff and Sutton, 2014).

Only electronic databases managed in English were selected. An electronic search of PubMed and Scopus databases from inception to 31 August 2021 was performed. Search strategies were conducted based on the following Medical Subject Headings (MeSH) terms: quality of life, post-traumatic stress disorder, mental health, nose neoplasms/psychology, and activities of daily living/psychology. These were then cross-referenced with the following terms: sinonasal tumor, sinonasal cancer, sinonasal neoplasm, head and neck tumor, head and neck cancer, head and neck neoplasm, and nose neoplasms.

The search terms used were as follows:

(“sinonasal tumor” OR “sinonasal cancer” OR” sinonasal neoplasm” OR “head and neck tumor” OR “head and neck cancer” OR “head and neck neoplasm” OR “Nose Neoplasms”) AND ((quality of life[mh]) or (Post traumatic stress disorder [mh]) or (Mental Health [mh]) OR (Nose Neoplasms/psychology[mh]) OR (Activities of Daily Living/psychology[mh])).

Although the search term “occupational etiology” was previously included, no research pertaining to a correlation with job-related factors or workplace exposures could be found. Therefore, this term was excluded.

Manual searches were performed for potentially eligible studies in the reference lists of previous reviews and included studies.

Two review authors independently and in duplicate screened titles and abstracts (phase 1) and then full texts (phase 2) of potentially relevant documents. A third review author resolved any disagreement between the two review authors. The selection of studies is presented in a flow diagram, according to the PRISMA guidelines (Liberati et al., 2009). The selected articles were then assessed for eligibility according to the following criteria.

### 2.1 Eligibility criteria

The Population, Exposure, Comparison, and Outcome (PECO) criteria (Morgan et al., 2018) are described below:

Types of population: the population suffering from a possible occupational hazard-related cancer.

Types of exposure: the population diagnosed with sinonasal, nasopharyngeal, and laryngeal cancers.

Types of comparators: comparators were not included.

Types of outcomes: the evaluation of the global quality of life, the emotional and social aspects of the quality of life of patients, and the assessment of anxiety and depression.

Studies were included if they (i) were original peer-reviewed research (qualitative or quantitative), (ii) described aspects of the psychological impact of the diagnosis of sinonasal and/or nasopharynx and/or laryngeal cancers, and (iii) reported results distinguished by tumor site (sinonasal, nasopharyngeal, and laryngeal). The psychological impact was referred in terms of the health-related quality of life, anxiety, and depression, detected by specific validated psychometric tools (questionnaires). The impact of diagnosis was considered to be measured at baseline (or pre-treatment time) and 1 year since diagnosis (to evaluate the variation in psychological impact over a short timeframe). This selection was based on the results of a study showing a high risk of suicidal tendencies in cancer patients, which is often caused by anxiety and depression. The study evidenced that, within 12 months after diagnosis, cancer patients still have twice the likelihood of dying by suicide (Saad et al., 2019).

The search was constrained by only including literature published in the English language and involving human participants. Studies were excluded if they did not report original research or were case reports or case series; included selected patient lists; involved genetic, cellular, or molecular studies; were non-longitudinal studies; and were commentaries, editorials, or review articles.

### 2.2 Assessment of risk of bias

For the risk of bias assessment, a validated tool was used to assess critical sources of bias, applying the navigation guide methodology (Woodruff and Sutton, 2014). The major domains of bias considered are as follows: selection, blinding, exposure, outcome, confounding, incomplete outcome data, selective outcome reporting, and conflict of interest. Each risk of bias domain was assigned a rating of “low,” “probably low,” “probably high,” or “high.” Two or more study authors independently assessed the risk of bias for each study. Where individual ratings differed, a third author resolved the conflict. For each included study, the risk of bias was reported at the individual study level per domain in a standard “Risk of Bias” table (Higgins et al., 2011).

## 3 Results

### 3.1 Study selection

**Figure 1:**
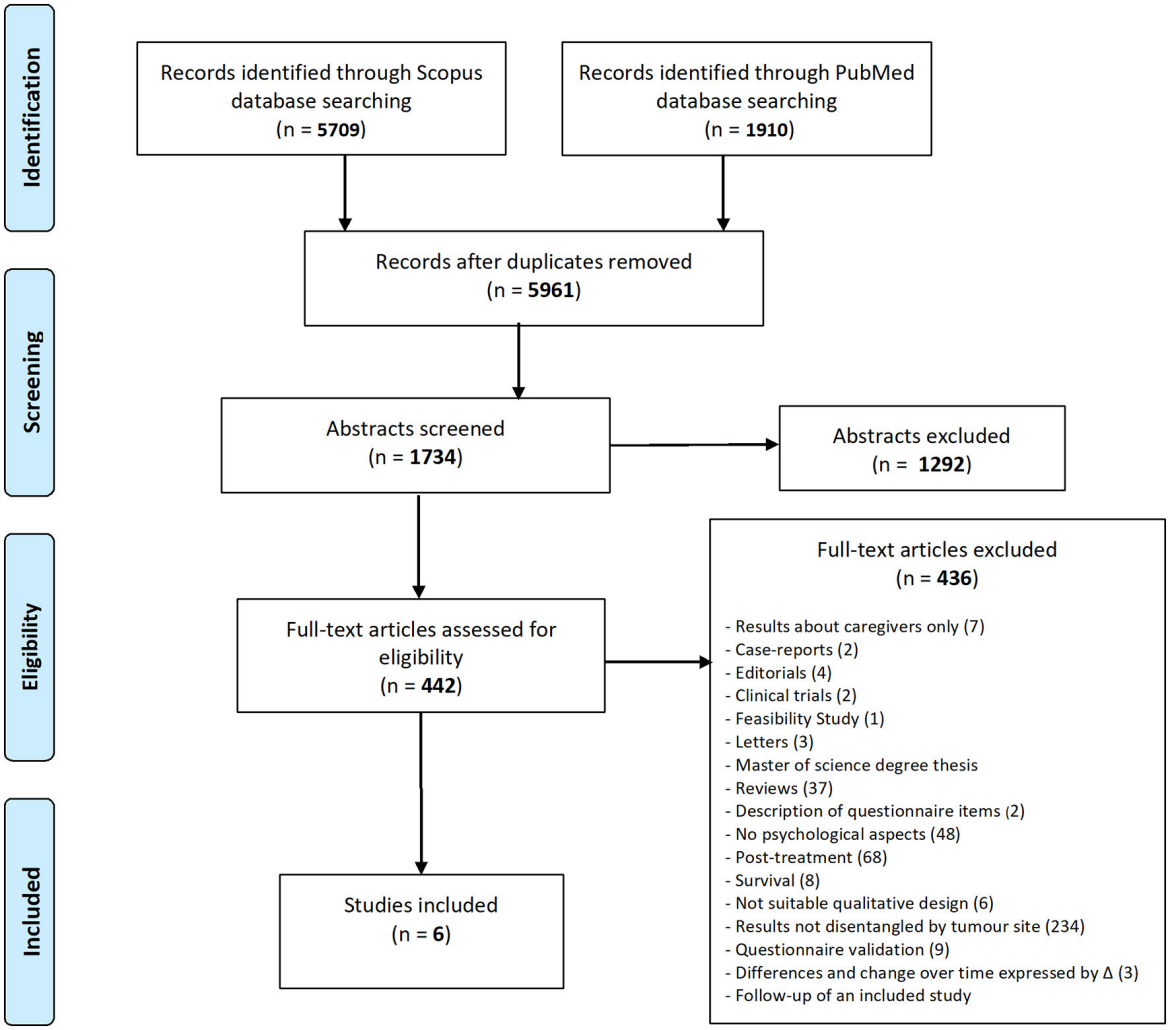
Flow diagram.

Out of the 5,961 records identified, 442 studies fulfilled the eligibility criteria. After full-text screening, we excluded 436 study records, leaving six studies (de Graeff et al., 2000; Hammerlid et al., 2001a,b; Finizia et al., 2002; Verdonck-de Leeuw et al., 2009; Sharma et al., 2019) (Figure 1). The six studies included were published in a 20-year timeframe, especially in the early years of this century. Three of these studies were conducted in Scandinavian countries, two in the Netherlands, and one in India. All studies conducted were longitudinal in nature, with psychological health assessments performed at the time of diagnosis in three of the studies and later but before treatment in the other three studies. Five of these studies reported results regarding quality of life associated with specific cancer sites. Furthermore, three studies reported on depression, two on both anxiety and depression, and one on mental distress (Table 1).

**Table 1:** Summary of relevant results of included studies (*N* = 6).

**References, country**	**Sample size (*N*)**	**Main psychological impact**	Characteristics of patients^*^: mean age (years, range), sex, and stage tumor	**Assessment of work aspects**	**Main results at diagnosis (score)**	**Main results at 1 year since diagnosis (score)**
Finizia et al. (2002), Sweden	26 laryngeal cancer cases	Assessment of health-related quality of life, anxiety, and depression	63 (26–76); male: 88%; female: 12%; stages: TI (50%), TII (19), TIII (12), TIV (12%); cancer *in situ* (8%)	Working (23%); Retirement pensioner (68%); disablement pension (15%); unemployed (4%)	*Larynx:* **EORTC QLQC30:** Emotional functioning: 77 Social functioning: 83 Global health status/QL: 65 **EORTC QLQH&N35**: Social contact: 8 **HADS**: Probable anxiety: 23%; possible anxiety: 15% Probable depression: 4%; possible: 15%	*Larynx (n.21):* **EORTC QLQC30:** Emotional functioning: 90 Social functioning: 90 Global health status/QL: 76 **EORTC QLQH&N35**: Social contact: 4 **HADS**: Probable anxiety: 0%; possible anxiety: 9.5% Probable depression: 4.7%; possible: 4.7%
Hammerlid et al. (2001a), Sweden and Norway	357 (86 laryngeal; 11 nasopharynx; 32 sinus and nose cancer cases)	Assessment of health-related quality of life	larynx: 66; nasopharynx: 53; sinus and nose: 68; male: 72%; female: 28%; stages TI-TII/TIII-TIV: 55/31 (larynx), 1/10 (nasopharynx) and 6/19 (sinus and nose)	30% employed, 6% unemployed	**EORTC QLQC30**: Emotional functioning: - Larynx: 76 - nasopharynx: 75 - sinus and nose: 75 Social Functioning: - larynx: 86 - nasopharynx: 68 - sinus and nose: 87 Global health status/QL: - larynx: 65 - nasopharynx: 58 - sinus and nose: 72 **EORTC QLQH&N35:** Social contact: - larynx: 7 - nasopharynx: 15 - sinus and nose: 7	EORTC QLQC30^**^: Emotional functioning: - larynx (n.60): 83 Social Functioning: - larynx (n.60): 86 Global health status/QL: - larynx (n.60): 69 **EORTC QLQH&N35:** Social contact: - larynx (n.60): 8
Hammerlid et al. (2001b), Sweden	232 (43 laryngeal cancer cases)	Assessment of health-related quality of life, anxiety, and depression	*Larynx:* 63 Male: 82% Female: 18% Stages TI-TII/TIII-TIV: 70%/30%	59% retired, 33% working, 6% unemployed; the rest of the patients were either studying or working from home	*Larynx:* **EORTC QLQC30:** Emotional functioning: 72 Social functioning: 83 Global health status/QL: 65 **EORTC QLQH&N35**: Social contact: 15	*Larynx:* **EORTC QLQC30:** Emotional functioning: 80 Social functioning: 82 Global health status/QL: 67 **EORTC QLQH&N35**: Social contact: 12 **HADS** Anxiety: Possible/probable anxiety: 1 year 27%; Depression: Possible/probable 1 year 27%
Verdonck-de Leeuw et al. (2009), The Netherlands	55 total (22 laryngeal/hypopharyngeal cancer cases)	Assessment of mental distress	63 (42–86) Male: 69%, Female: 31% Stages: TI (36%), TII (24%), TIII (24%), TIV (16%)	No	*Larynx/hypopharynx* **HADS** High level of distress (total HADS score >15): 5% (*p* < 0.01)	*Larynx/hypopharynx* HADS^***^ High level of distress (total HADS score >15): 32% (*p* < 0.01)
de Graeff et al. (2000), The Netherlands	107 patients (46 laryngeal cancer cases)	Assessment of health-related quality of life and depression	60 (31–73) Male: 80%, female: 20% Stages: T0 (2%), TI (42%), TII (22%), TIII (12%), and TIV (22%)	No	*Larynx:* **EORTC QLQ-C30(+3)**: Emotional functioning: 69.6 Social functioning: 86.3 Global quality of life: 73.5 **EORTC H&N35** Social contact: 4 **CES-D**: Total score: 12.7 Percentage with total score ≥16: 28.9	*Larynx:* **EORTC QLQ-C30(+3)**: Emotional functioning: 80.2 Social functioning: 93 Global quality of life: 79.8 **EORTC H&N35** Social contact: 4 **CES-D**: Total score: 9.8 Percentage with total score ≥16: 15.6
Sharma et al. (2019), India	130 patients (13 laryngeal cancer cases)	Assessment of health-related quality of life	6 (age-group: 20–35 years); 65 (age-group 36–50 years); 59 (>50 years) male: 80%, female: 20% stages: TI (20%), TII (31.5%), TIII (13.1%), TIV (35.4%)	No	*Larynx:* **FACT:** Emotional domain of quality of life: 20.23 (min–max: 18–22) Social domain of quality of life: 23.85 (min–max: 23–26)	*Larynx:* **FACT:** Emotional domain of quality of life: 17.15 (min–max: 12–22) Social domain of quality of life: 22.15 (min–max: 21–23)

^*^If not specified, data refer to the whole sample size.

^**^Data referring to the study of Bjordal et al. (2001) that is the follow-up of the study by Hammerlid et al. (2001a).

^***^Median time since diagnosis = 4.2 months.

### 3.2 Patient characteristics

The vast majority of patients enrolled in these studies were affected by laryngeal cancer (*n* = 236), which was investigated in all studies and represented 85% of all patients, while patients affected by sinonasal (*n* = 32) and nasopharyngeal cancers (*n* = 11) were fewer, with each being reported in only one study. Male patients were predominant in all of these studies, reflecting the higher incidence of head and neck cancers among male gender, with proportions ranging from 69 to 88% in the different studies. The mean age of the patients was mostly 70 years, except for the Indian study, where more than half the patients were younger than 50 years (Sharma et al., 2019). The majority of patients with laryngeal cancer were in tumor stage I or II, whereas greater proportions of patients with the other two cancer types were in stage III or IV. Data on employment status, collected in three studies, revealed that less than one-third of the patients enrolled were still working at the time of the study.

### 3.3 Outcome assessment tools

Quality of life was assessed in four out of five studies using both the general EORTC QLQ-30 questionnaire and the specific EORTC QLQ-H&N35 questionnaire for head and neck cancers; however, in the Indian study, a different questionnaire was used (FACT questionnaire for head and neck cancer).

The EORTC QLQ-C30 (Fayers et al., 2001) is a cancer-specific questionnaire and comprises a global HRQoL scale and five functional scales: physical functioning, role functioning, emotional functioning, cognitive functioning, and social functioning. There are three symptom scales (fatigue, nausea and vomiting, and pain) and six single items relating to dyspnea, insomnia, loss of appetite, constipation, diarrhea, and financial difficulties. During the scoring procedure, raw EORTC QLQ-C30 scores are linearly transformed to 0 and 100 scales. For global health status and the five functioning scales, a score of 100 indicates a high HRQoL. On the other hand, for financial difficulties and the eight symptoms, a score of 100 implies the highest level of difficulty or symptom burden.

The EORTC QLQ-HN35 module (Fayers et al., 2001) addresses specific issues related to head and neck cancers and comprises seven subscales: pain, swallowing, senses, speech, social eating, social contact, and sexuality. There are 10 single items that address specific problems: issues with teeth, dry mouth, sticky saliva, cough, difficulty in opening the mouth wide, weight loss, weight gain, use of nutritional supplements, use of feeding tubes, and use of painkillers. The scores of the QLQ-HN35 are linearly transformed to a scale of 0–100, with higher scores corresponding to greater perceived functional impairment.

The Functional Assessment of Cancer Therapy—Head & Neck (FACT-HN) (D'Antonio et al., 1996; List et al., 1996) is a multidimensional, self-reported quality of life instrument specifically designed for use with head and neck cancer patients. It consists of 27 main items—FACT-General (FACT-G)—that assess patient function in four domains: physical, social/family, emotional, and functional wellbeing. It is further complemented by 12 site-specific items for the assessment of head and neck-related symptoms—FACT—(H&N-G). Each item is rated on a 4-point Likert scale and then combined to produce subscale scores for each domain as well as an overall HRQoL score. A subscale score is computed by summing across all items, with higher scores reflecting a better QOL. For the domains “Emotional Well Being (EWB)” and “Social/family Well Being (SWB),” the score ranges are 0–24 and 0–28, respectively.

The two studies which assessed both anxiety and depression employed the Hospital Anxiety and Depression Scale (HADS) questionnaire, while the one that assessed only depression used the CES-D questionnaire. The level of distress was also assessed through the HADS questionnaire in one study.

The HADS (Spinhoven et al., 1997; Walker et al., 2007) is a 14-item questionnaire with two subscales, anxiety and depression. The total HADS score ranges from 0 to 42, while the subscales range from 0 to 21. For anxiety and depression, possible cases are defined as having a subscale score of 8–10, whereas probable cases indicate a score higher than 10. A total HADS score higher than 15 corresponds to a high level of psychological distress.

The CES-D Scale (Radloff, 1977; Orme et al., 1986) is an instrument used for measuring depression in the general (non-psychiatric) population. The total score of the CES-D Scale ranges from 0 to 60, with higher scores reflecting a higher level of depression. Patients with a score of 16 or more are classified as having significant depressive symptomatology.

### 3.4 Quality of life and mental health at diagnosis

From the six studies selected, the scores for the EORT QLQ-30, FACT, and EORT QLQ-H&N35 indicated that the QoL at the time of diagnosis was similar to that in the general population, remaining constant or slightly improving at 1 year since diagnosis. In contrast, the manifestation of anxiety and depression was stronger at diagnosis, although one study revealed a higher percentage at 1 year post-diagnosis (total HADS score >15). No study examined the correlation between the psychological impact of cancer diagnosis and its possible occupational etiology (Table 1).

Regarding the dimensions of QoL assessed through the questionnaire EORTC QLQ-30, mean scores of emotional functioning at diagnosis were in the range 70–79 and were similar across cancer sites. These values correspond to an average emotional impact on the quality of life of these patients; in fact, the values are comparable to the average of the normative head and neck sample (72.5) (Aaronson et al., 1993; Fayers et al., 2001). The social functioning scores were all above 80, in line with the average of the normative head and neck patient sample (82.6), with an exception for patients with nasopharyngeal cancer, for whom a lower score was reported in one study (score = 68), corresponding to a lower social quality of life (Hammerlid et al., 2001a).

The mean scores for social contact, a dimension assessed through the EORTC QLQ-H&N35 questionnaire, varied widely (range: 4–15), with three out of four studies showing values below 10, suggesting a low significance of the problem.

The global health scores were also consistent with the average of the normative reference sample (64.1), except for patients with nasopharyngeal cancer (score = 58) (Hammerlid et al., 2001a), for whom a lower global health score was observed.

In the Indian study where quality of life was assessed using the FACT questionnaire, higher than average normative reference scores (List et al., 1996) were observed in both the emotional (score = 20.2) and social domains of quality of life (score = 23.9), suggesting a slightly better quality of life. For both scales, possible values ranged from 0 to 28 (Sharma et al., 2019).

In the only study which assessed anxiety and depression using the HADS questionnaire, the prevalence of probable/possible anxiety was 38% at diagnosis and that of probable anxiety was 23%; corresponding figures for depression were 19% for possible/probable depression and 4% for probable depression (Finizia et al., 2002). In the study which assessed depression using the CES-D questionnaire, 28.9% of subjects had a score above 15, which is the criterion for indicating the presence of depression (de Graeff et al., 2000). A Dutch study using the HADS questionnaire found a low prevalence of high mental distress, defined as a HADS score higher than 15, among a series of 22 larynx/hypopharynx patients (5%) (Verdonck-de Leeuw et al., 2009).

### 3.5 Quality of life and mental health at 1 year after diagnosis

In most studies, scores of the social and global EORTC QLQ-30 scales remained similar to the general population or slightly increased after 1 year from diagnosis, while a more marked improvement was observed for the emotional domain (range 80–90). In contrast, in the only study using the FACT questionnaire, there was a worsening of the QoL in the emotional (score = 17.15) and social domains (score = 22.15) (Sharma et al., 2019).

The results of the dimension of social contact, assessed using the EORTC QLQ-H&N35 questionnaire, were largely consistent with those from the social dimension scale of the EORTC QLQ-30, although widely variable across studies. This finding showed a substantial improvement of this dimension in two out of four studies (Hammerlid et al., 2001b; Finizia et al., 2002), while the other two studies indicated a stability of the score (de Graeff et al., 2000; Hammerlid et al., 2001a).

Regarding mental health, one study utilizing the HADS questionnaire found a decrease in the proportion of patients affected by possible/probable anxiety and possible depression, while the incidence of probable depression remained unchanged (Finizia et al., 2002). The study that used the CES-D questionnaire also found a decrease in the depression score and in the proportion of subjects with severe depression (CES-D score ≥16) (de Graeff et al., 2000). In contrast, the proportion of subjects with high levels of mental distress strongly increased at 1 year from diagnosis in another study (from 5 to 32%) (Verdonck-de Leeuw et al., 2009). High prevalence rates of both possible/probable anxiety and depression (27% for both: the authors do not distinguish possible and probable cases), measured using the HADS questionnaire, were also identified in a Swedish study during 1-year follow-up; however, it was not possible to compare these findings with those at diagnosis, as the authors did not report them (Hammerlid et al., 2001b).

### 3.6 Risk of bias

The risk of bias was rated based on the information available in the included studies. The risk of bias ratings for each domain for all the six included studies are reported in Table 2. With regard to “selection,” the risk was rated as low for one study and probably low for five studies. We evaluated the risk of bias as low because the criteria for selecting populations were sufficiently detailed and data were supplied exhaustively. The “probably low” risk of bias was assigned because the studies provided indirect evidence that their sample criteria were adequately described. For the domain “blinding,” the risk of this bias was rated as low for all six studies: although this bias is not very appropriate to these types of studies, we cannot rule out bias (it is possible that interviewers were aware of case severity). In addition, for “exposure,” the rating was low risk of bias because all the studies provided adequate accuracy in identifying the health status of selected patients. With respect to the “outcome,” the risk of this bias was evaluated as low for one study and probably low for five studies. All studies used the standardized methods for assessing quality of life, i.e., questionnaires previously validated. The risk of “confounding” was graded as low for three studies, probably low for one study, and probably high for two studies. A low risk of bias was attributed because the studies appropriately stratified results by sex, age, tumor stage, and separately for each tumor site. A probably low risk was assigned since the control for confounders was applied to the whole sample, without stratifying it by tumor site. Conversely, a probably high risk of bias was attributed because the studies assessed a portion of the crucial confounding factors, but not all. Regarding “incomplete outcome data,” the risk of bias was rated as probably low for five studies and probably high for one study. The outcome measurements were obtained by evaluating answers to the submitted questionnaires: in five studies, there was not sufficient information provided, but the reasons were thoroughly examined, although not all within the sixth study. Bias due to “selective outcome reporting” was evaluated as low risk for all six studies because the Results section included the outcomes as detailed in the Methods section. The risk of “conflict of interest” was graded as low in all six studies, as we were unsuccessful in finding the indication of a conflict of interest declared by the authors.

**Table 2:** Overview of the risk of bias assessment.

**References**	**Journal**	**1) Selection**	**2) Blinding**	**3) Exposure**	**4) Outcome**	**5) Confounding**	**6) Incomplete outcome data**	**7) Selective outcome reporting**	**8) Conflict of interest**
Finizia et al. (2002)	*Acta Oncologica*	2	1	1	2	3	2	1	1
Hammerlid et al. (2001a)	*The Laryngoscope*	2	1	1	2	3	2	1	1
Hammerlid et al. (2001b)	*Head & Neck*	2	1	1	2	2	2	1	1
Verdonck-de Leeuw et al. (2009)	*Oral Oncology*	2	1	1	1	1	2	1	1
de Graeff et al. (2000)	*The Laryngoscope*	1	1	1	2	1	2	1	1
Sharma et al. (2019)	*Indian J Otolaryngol Head Neck Surg*	2	1	1	2	1	3	1	1

Evaluation: 1 = low; 2 = Probably Low; 3 = Probably High.

## 4 Discussion

This review intended to highlight the psychological impact of SNC, NPC, and laryngeal cancer diagnosis associated with the possible occupational etiology of these neoplasms and to explore the instruments that evaluate QoL, anxiety, and depression in these patients.

Only six studies met the eligibility criteria. Three studies provided limited information about employment status of the patients, but outcomes of QoL and distress were never associated with occupational characteristics.

The selected studies mainly included cases of laryngeal cancers. A very small number of patients affected by SNC and NPC were examined. Therefore, the results for these cancer types should be interpreted with caution, as they may have been affected by sampling variability. Moreover, the identified studies were designed in the mid-to-late 1990–2000s and may not reflect possible improvements in the therapies.

The EORTC QLQ-C30, EORTC QLQ-H&N35, and FACT questionnaires to assess the QoL and the HADS and CES-D questionnaires to evaluate anxiety and depression were identified in this review. These tools will be considered for future studies on the topic, although a review by Shunmugasundaram et al. highlights that the best tools to assess anxiety and depression in HNC patients are “The Patient Health Questionnaire-9, Zung Self-rating Depression, and Zung Self-rating Anxiety Scales” (Shunmugasundaram et al., 2020).

Regarding laryngeal cancers, the EORTC QLQ scores were consistent with the normative values of head and neck cancers at diagnosis (Fayers et al., 2001).

Normative values of EORTC QLQ-C30 were investigated in the general population of 15 countries in Europe and North America (Nolte et al., 2019). In terms of emotional functioning, in Swedish general population, the mean value was 76.7, comparable to those found in the present review by Hammerlid et al. (2001a,b) and Finizia et al. (2002) at diagnosis, which further improved at 1 year post diagnosis. In the Netherlands, the normative values were higher than those found by de Graeff et al. (2000), although these improved at 1 year post diagnosis, as it has also been observed in the Swedish studies. Therefore, the emotional impact appears more relevant at diagnosis than 1 year later (Hammerlid et al., 1999; Ronis et al., 2008).

The values of social functioning in the Swedish studies included in our review were slightly lower than the data for the same country (91.4), as found by Nolte et al. (2019) both at diagnosis (Hammerlid et al., 2001a,b; Finizia et al., 2002) and 1 year later, particularly for the nasopharynx (Hammerlid et al., 2001a). Similarly, the observed value in the Dutch study (de Graeff et al., 2000) was lower than the one reported by Nolte et al. (2019) (91.9).

Regarding global health status, the values in the Swedish and Dutch studies are comparable with the normative ones, except for the nasopharynx, which is much lower (Hammerlid et al., 2001a). At 1 year since diagnosis, values tended to be higher.

The QoL in the Indian study (Sharma et al., 2019) was evaluated using the FACT questionnaire, and the values of both emotional and social domains at diagnosis were higher than average normative reference scores (List et al., 1996), indicating that the QoL was preserved at diagnosis, but the emotional aspect worsened at 1 year since diagnosis.

In relation to the assessment of anxiety, our results indicate instead a higher prevalence, compared to the general population: in the Swedish study by Finizia et al. (2002), possible/probable anxiety was found to affect more than 30% of cancer patients and to decline 1 year since diagnosis to values similar to those of the general Swedish population (Lisspers et al., 1997). In addition, the proportion of the affected subjects in the study considered was more than double the reported rates for probable anxiety (HADS score higher than 10) in the general populations of Sweden (8%) (Lisspers et al., 1997), UK (10%) (Crawford et al., 2001), or Germany (men: 5.4%; women: 8.5%) (Hinz and Brähler, 2011).

For depression, a higher prevalence was found among laryngeal cancer patients compared to the Swedish general population, with reported proportions of possible/probable depression being ~8%−10% (Lisspers et al., 1997; Djukanovic et al., 2017). Similar results were observed in the studies conducted in other countries (Crawford et al., 2001; Nortvedt et al., 2006; Grav et al., 2012), although the study by Breeman et al. (2015) on the general Swedish population found a proportion of ~15% in both genders (Breeman et al., 2015). In the Dutch study assessing depression through the CES-D questionnaire (de Graeff et al., 2000), the percentage of laryngeal cancer patients with a total score ≥16 was 28.9% at the time of diagnosis, indicating a significant depressive symptomatology (Orme et al., 1986), and nearly, i.e., 15.6%, at 1 year since diagnosis. The prevalence at the time of diagnosis (28.9%) was more than double the rate of prevalence found in a sample of the healthy general Dutch population (12.5%), as determined using the same CES-D questionnaire with a cutoff score of 16 (Bouwman et al., 2010).

In contrast, the study by Verdonck-de Leeuw et al. (2009) reported a prevalence of high levels of distress (total HADS score >15) that was more marked at 1 year since diagnosis (32%) than at the time of diagnosis (5%), possibly as a result of the treatment's side effects, although other factors such as the stage and location of the tumor, any underlying distress, and individual personality traits can be reasonably involved. A similar prevalence of high levels of distress at 1 year since diagnosis (27% for both probable/possible anxiety and depression) was found in the study by Hammerlid et al. (2001b) (data not reported at diagnosis).

These results are consistent with those of a study that analyzed the prevalence of psychological distress among a large sample of ~4,500 cancer patients, which suggested that the prevalence rate of distress for head and neck tumors was 35.1%. This rate varied from 43.4% for lung cancer to 29.6% for gynecological tumors (Zabora et al., 2001).

Globally, psychological stressors associated with head and neck cancers mainly consist of uncertainty, obstacles to activities and communication, fear of recurrence, apprehension for disease and treatment, and the expected negative surgical consequences on the body aspects. The main challenge for these cancer patients indeed is their facial disfigurement. This aspect is more evident for SNCs, where the face can be seriously compromised by surgical interventions, implying considerable difficulties in managing their life. Many interviewers, involved in submitting the ReNaTuNS questionnaire, referred a deep psychological distress in the patients, often leading to the refusal of the interview. Suffering from facial deformity because of such type of cancers implies experiencing deep psychological trauma associated with a loss of self-esteem and awareness of limited attractiveness (Moadel et al., 1998). Moreover, decreased feelings of sexuality associated with humiliation and increased isolation have also been reported (Curtis and Zlotolow, 1980; Meyers et al., 1980) as well as greater social isolation (Strauss, 1989; Gritz et al., 1999). The most adopted coping strategies at diagnosis such as denial, behavioral disengagement, and self-blame were predictive of post-traumatic stress disorder symptoms and poor quality of life (Richardson et al., 2016). Regarding personality characteristics, the results of a review (Llewellyn et al., 2005) showed that the higher the patient's extraversion or optimism score, the higher his health-related quality of life (HR-QoL) score. In contrast, patients with a high score for neuroticism were more likely to have a low HR-QoL.

Psychological consequences resulting from illness or treatment have been observed in long-term cancer survivors (Brandenbarg et al., 2019), although often underdiagnosed and undertreated. They experience greater anxiety, pain, fatigue, psychological and social impacts (fear, alienation, and disfigurement), and feelings of guilt for lifestyle choices that may have impacted the cancer risk.

Although no suicide case was identified in the present review, all these stressors are considered reasons for the elevated risk of suicide observed in cancer patients. A study observed that the risk remains elevated in the first 6 months after diagnosis, with a suicide rate ~2.5 times higher than the expected rate in the general population (Saad et al., 2019).

In an effort to comprehend and cope with their condition, individuals diagnosed with cancer may formulate beliefs about the origin of their illness, and these attributions may influence their psychosocial adaptation. A review about causal attributions for breast cancer revealed a consistent belief among survivors that their breast cancer could be linked to family history, environmental factors, stress, fate, or chance (Dumalaon-Canaria et al., 2014). Comprehending and evaluating causal attributions and existential questions related to diagnosis can significantly enhance understanding of survivors' adaptation and psychosocial wellbeing (Ferrucci et al., 2011). Causal attributions may also include the occupational environment, and this aspect can impact cancer patients' quality of life and distress levels.

Not having specific data on work-related aspects related to emotional distress and quality of life represents a significant limitation, which should be addressed in further studies. The only work-related aspect considered is the return to work after the diagnosis and treatment of head and neck cancer patients. The percentage of those who were still working varies from 40% at 12 months post-diagnosis to over 80% 2 years after diagnosis (Buckwalter et al., 2007; Verdonck-de Leeuw et al., 2010). Furthermore, some of those who return to work tend to reduce the duration of their working hours (Harrison et al., 1997), change tasks, or change jobs (Verdonck-de Leeuw et al., 2010; Chang et al., 2012). Survivors of head and neck cancers express their desire for more information on returning to work during their health consultations, but they perceive it to be absent (Miller et al., 2023). Returning to work depends on the type of cancer and the specific treatment received. Head and neck cancer survivors were more likely to remain on sick leave at the beginning of the recovery period in comparison with breast cancer patients (So et al., 2022). Moreover, a study found that head and neck cancer survivors who experienced stronger negative impacts from their cancer took longer to return compared to breast, gynecological, and urological cancer survivors (Cooper et al., 2013).

A major strength of our review is that we included studies that presented data on the QoL and prevalence of anxiety and depression in SNC, NPC, and laryngeal cancer, both at diagnosis and 1 year later. On the basis of the results, the most relevant impact is represented by anxious and depressive experiences mainly at diagnosis. Focusing on symptoms' prevalence in these patients is essential for early intervention programs to address specific healthcare needs and treatments. Moreover, the review has highlighted the different questionnaires used for outcome assessment.

This review has the following limitations: the partial use of databases (only Scopus and PubMed were used) and the exclusion of gray literature. Moreover, only longitudinal studies were found, which are more vulnerable to bias due to unmeasured confounding factors. Moreover, most studies investigated QoL, and psychological health in head and neck cancers were not distinguished by the specific site of the tumor.

This review revealed a clear aspect of distress characterized by anxiety and depression at the time of diagnosis, which must be considered in a holistic perspective. This psychological suffering can also have peculiarities related to past/current exposure to occupational carcinogenic hazards since it is a group of cancers in which the occupational etiological fraction is high, and this aspect is not considered in the scientific literature.

## 5 Conclusion

The diagnosis of a head or neck tumor can have a significant impact on mental health, psychological distress, and quality of life of patients. It is important that they have access to comprehensive support not only for medical treatment but also for psychological and social assistance. Interventions such as individual or group therapy can help patients manage emotional stress, cope with challenges related to the illness, and improve their quality of life during and after treatment. Additionally, involvement of family members and caregivers is crucial to ensure adequate emotional and social support for patients. When patients' clinical history is collected, their employment history must be systematically investigated and the possible consequences of the awareness of an occupational etiology in the evaluation of the psychological distress also need to be considered. We suggest addressing these concerns in the design of future study protocols.

## Data availability statement

The original contributions presented in the study are included in the article/supplementary material, further inquiries can be directed to the corresponding author.

## Author contributions

MB: Writing – original draft, Writing – review & editing. Ad'E: Writing – review & editing. SR: Writing – review & editing. CM: Writing – review & editing. LM: Writing – review & editing. RC: Writing – review & editing. RG: Writing – review & editing. SP: Writing – review & editing. PM: Writing – review & editing. DV: Writing – review & editing. AB: Writing – original draft, Writing – review & editing.
